# Changes in anterior segment parameters following insertion of ExPRESS mini glaucoma implant vs. trabeculectomy

**DOI:** 10.5935/0004-2749.20200040

**Published:** 2020

**Authors:** Eitan Livny, Na’ama Hammel, Michael Mimouni, Moshe Lusky, Igor Kaiserman, Irit Bahar

**Affiliations:** 1 Departmens of Ophthalmology, Rabin Medical Center, Petah Tikva, Israel; 2 Sackler Faculty of Medicine, Tel Aviv University, Tel Aviv, Israel; 3 Department of Ophthalmology, Rambam Health Care Campus, Haifa, Israel; 4 School of Medicine, Technion - Israel Institute of Technology, Haifa, Israel; 5 Department of Ophthalmology, Barzilai Medical Center, Ashkelon, Israel; 6 Faculty of Health Sciences, Ben-Gurion University of the Negev, Beer-Sheba, Israel

**Keywords:** Glaucoma/surgery, Glaucoma drainage implants, Trabeculectomy/methods, Intraocular pressure, Glaucoma/cirurgia, Implantes para drenagem de glaucoma, Trabeculectomia/métodos, Pressão intraocular

## Abstract

**Purpose:**

To compare changes in anterior segment parameters following ExPRESS Mini Glaucoma Shunt surgery vs. trabeculectomy using the Pentacam rotating Scheimpflug camera.

**Methods:**

In this prospective, comparative study, 27 patients with glaucoma treated at the Rabin Medical Center from 2009 to 2013 were enrolled in this prospective comparative study: 19 participants (19 eyes) underwent ExPRESS shunt implantation and 12 (13 eyes) underwent trabeculectomy. Changes in anterior chamber parameters at postoperative day 1 and postoperative month 3 were evaluated on Scheimpflug images.

**Results:**

Intraocular pressure decreased significantly from baseline in both groups. The decrease in both groups was similar at postoperative month 3 (p=0.82). ExPRESS surgery caused a transient increase in posterior corneal astigmatism (p=0.008) and a transient decrease in anterior chamber depth (p=0.016) and volume (p=0.006) on postoperative day 1. At postoperative month 3, these parameters were no longer statistically significant (p=0.65, p=0.51, and p=0.57 respectively). Trabeculectomy caused a transient increase in anterior and posterior corneal astigmatism on postoperative day 1 (p=0.003 and p=0.005, respectively), which were not evident at postoperative month 3 (p=1.0 and p=1.0, respectively). At postoperative month 3, both ExPRESS and trabeculectomy showed similar changes in anterior chamber parameters.

**Conclusions:**

Both ExPRESS mini glaucoma implant and trabeculectomy significantly decreased intraocular pressure and had transient effects on anterior segment parameters, with minor differences between the methods.

## Abstract (pt)

**Objetivo:**

Comparar as alterações nos parâmetros do segmento anterior após a cirurgia ExPRESS Mini Glaucoma Shunt vs. trabeculectomia usando a câmera Scheimpflug Pentacam rotativa.

**Métodos:**

Neste estudo comparativo prospectivo, 27 pacientes com glaucoma tratados no Centro Médico Rabin de 2009 a 2013 foram incluídos neste estudo comparativo prospectivo: 19 participantes (19 olhos) foram submetidos ao implante de derivação ExPRESS e 12 (13 olhos) foram submetidos à trabeculectomia. Alterações nos parâmetros da câmara anterior no dia 1 e em 3 meses de pós-operatório foram avaliadas pelas imagens de Scheimpflug.

**Resultados:**

A pressão intraocular diminuiu significativamente em relação aos valores iniciais nos dois grupos. A diminuição nos dois grupos foi semelhante no 3º mês pós-operatório (p=0,82). A cirurgia com ExPRESS causou um aumento temporário do astigmatismo posterior da córnea (p=0,008) e uma diminuição temporária da profundidade da câmara anterior (p=0,016) e do volume (p=0,006) no primeiro dia do pós-operatório. Ao final de três meses, esses parâmetros não foram mais estatisticamente significativos (p=0,065, p=0,51 e p=0,57, respectivamente). A trabeculectomia causou um aumento temporário do astigmatismo anterior e posterior da córnea no primeiro dia do pós-operatório (p=0,003 e p=0,005, respectivamente), mas isso não foi observado ao final de 3 meses (p=1,0 e p=1,0, respectivamente). Após 3 meses, tanto o EXPRESS quanto a trabeculectomia mostraram alterações semelhantes nos parâmetros da câmara anterior.

**Conclusões:**

O implante ExPRESS Mini para glaucoma e a trabeculectomia diminuíram significativamente a pressão intraocular e tiveram efeitos temporários nos parâmetros do segmento anterior, com pequenas diferenças entre os métodos.

## INTRODUCTION

The ExPRESS^TM^ Mini Glaucoma Shunt (Alcon Laborat ories, Inc., Fort Worth, TX, USA) is a non-valved, flow-restricting, biocompatible, stainless steel device used for intraocular pressure (IOP) lowering surgery. The device was originally designed for implantation under the conjunctiva as an alternative to trabeculectomy^(1,2)^, a surgical technique that can result in numerous complications, including hypotony, erosion, and extrusion of the device^(3-8)^. In 2005, Dahan and Carmichael proposed implantation under a scleral flap in order to reduce the rate of complications associated with placement under the conjunctiva^(9)^. This technique has almost completely eliminated the erosion complication and has been reported to result in lower rates of hypotony than trabeculectomy^(9-13)^. The ExPRESS shunt offers a small, more precise, and controlled surgical wound with more predictable and reliable results, while avoiding the need for iridectomy^(1,13-16)^. In addition, some evidence suggests that vision may recover more rapidly after ExPRESS implantation^(13,14,16-18)^.

In a previous study, we evaluated the effects of the ExPRESS shunt on corneal curvature and anterior segment parameters using images captured with a Pentacam rotating Scheimpflug camera (Oculus Optikgeräte GmbH, Wetzlar, Germany)^(19)^. The results showed a sig nificant reduction in IOP and a transient effect on anterior segment parameters. The purpose of the present study was to compare these findings with the effects of trabeculectomy on the same parameters, as evaluated using Scheimpflug images.

## METHODS

### Participants

The study participants were recruited from the Glaucoma Clinic of the Rabin Medical Center (Petah Tikva, Israel) between 2009 and 2013. All participants underwent IOP-lowering treatment due to uncontrolled IOP by means of topical medications and/or a glaucoma laser procedure. Prior to surgery, all patients underwent a complete ophthalmologic examination, which included best-corrected visual acuity (BCVA), slit-lamp biomicroscopy, including IOP measurement using Goldmann applanation tonometry, and dilated fundoscopy. All patients provided written informed consent and were older than 18 years. The study protocol was approved by the Ethics Review Board of the Rabin Medical Center and was conducted in accordance with the tenets of the Declaration of Helsinki.

### Primary open angle diagnosis

Criteria for a diagnosis of primary open angle glaucoma (POAG) were an open angle and glaucomatous appearance of the optic nerve head with a correlating typical glaucomatous visual field loss. Primary filtration surgery was indicated in each participant, all of whom were on maximal tolerated ocular hypotensive medications and did not reach the target IOP, as measured with a Goldmann applanation tonometer.

### Instrumentation

Images of the cornea were taken using a Pentacam rotating Scheimpflug camera. Pentacam images of the cornea were taken prior to surgery, postoperative day (POD) 1, and postoperative month (POM) 3. Twenty-five single images, separated by 7.2º, were captured by the rotating camera in less than 2 s. Repeated imaging was performed with the Pentacam until sufficient quality was achieved. All images were manually reviewed to validate correct surface identification. Findings of the anterior and posterior corneal curvature, anterior and posterior corneal astigmatism, and anterior chamber depth (ACD), volume (ACV), and angle (ACA) were compared over time and between groups.

### Surgery

It is routine in our department to perform cataract sur gery prior to trabeculectomy for any patient with clinically significant cataract as a two-stage procedure. All surgeries were performed by one of two experienced surgeons in a similar fashion, as described herein. Surgery was performed under sub-Tenon’s anesthesia. A fornix-based conjunctival flap was created and a sponge soaked with 0.04% mitomycin C was applied under the conjunctival flap for 1 to 2 min, depending on the individual risk of scarring, and then rinsed. ExPRESS surgery was performed as described by Dahan and Carmichael^(9)^. In brief, a partial thickness scleral flap was created and the ExPRESS shunt (P-50 model) was inserted into a tract formed using a 25 gage needle mounted on a 5-mL syringe. Scleral and conjunctival flaps were sutured using interrupted 9-0 nylon sutures. Trabeculectomy was performed using releasable sutures as described by Kolker et al.^(20)^. In brief, a partial thickness scleral flap was dissected, followed by trabeculectomy using a Kelly decsement’s punch. Iridectomy was performed and the scleral flap was secured with releasable sutures. The conjunctiva was then sutured with 9-0 nylon. Postoperatively, all patients received topical prednisolone acetate every 2 h for one week with tapering down over a period of 6-8 weeks and topical antibiotics four times daily for one week. Patients were instructed to discontinue IOP-lowering medications following surgery. All patients were followed-up for a minimum of 3 months.

### Statistical analysis

All analyses were performed with SPSS software, version 12 (SPSS, Inc., Chicago, IL, USA). The normality of data was tested using visual (histograms and probability plots) and analytical methods (Kolmogorov-Smirnov/ Shapiro-Wilk test). The paired *t*-test and Wilcoxon signed-rank test were used to compare paired preoperative and postoperative variables. The Student’s *t*-test was used to compare changes in parameters between groups at the final endpoint (3 months). Bonferroni correction was used for multiple comparisons at different time points. Assuming a preoperative and postoperative ACD of 4.3 ± 0.7 and 3.5 ± 1 mm, respectively, at a power of 0.80 in the ExPRESS group, the calculated minimum sample size was approximately n=10^(19)^. When this pilot study was conducted, there were no previous data or reports regarding changes in ACD following trabeculectomy as evaluated by Scheimpflug images, thus we assumed that at least 10 subjects would be required in this group as well.

## RESULTS

Nineteen consecutive patients underwent surgery with the ExPRESS Mini Glaucoma Shunt (ExPRESS group, 19 eyes) and 12 underwent traditional trabeculectomy (trabeculectomy group, 13 eyes). No patient dropped out during the study period.

There were no significant differences in age distribution (p=0.81), sex (p=0.21), or type of glaucoma (p>0.1) between the ExPRESS and trabeculectomy groups (Table 1). The main diagnosis was POAG (42.1% and 42.6% in the ExPRESS and trabeculectomy groups, respectively). All patients in the ExPRESS group and six in the trabeculectomy group underwent previous phacoemulsification surgery.

**Table 1:** Clinical characteristics of glaucoma patients treated with the ExPRESS implant or trabeculectomy

Parameter	express	Trabeculectomy	p-value
Age, years (mean±SD)	71.4 ± 15	72.5 ± 9.0	0.810^*^
Sex (male/female), n	12/7	4/8	0.210^**^
Diagnosis			
POAG	8 (42.1%)	6 (46.2%)	0.640^**^
PEX glaucoma	6(31.6%)	2 (15.4%)	0.530^**^
NVG	3 (15.8%)	2 (15.4%)	0.890^**^
Uveitic glaucoma	2 (10.5%)	0 (0%)	0.580^**^
CNAG	0 (0%)	3 (23.1%)	0.110^**^
Surgical history			
Phacoemulsification	19 (100%)	6 (46.2%)	0.007^**^
Trabeculectomy	9 (47.4%)	0 (0%)	0.010^**^
Pars plana vitrectomy	3 (15.8%)	0 (0%)	0.370^**^
PKP	2 (10.5%)	0 (0%)	0.640^**^
express	1 (5.3%)	0 (0%)	0.850^**^
Intravitreal bevacizumab injection	0 (0%)	1 (7.7%)	0.840^**^
ALT	0 (0%)	1 (7.7%)	0.840^**^
Laser iridotomy	0 (0%)	1 (7.7%)	0.840^**^

ALT= argon laser trabeculoplasty; CNAG= chronic narrow angle glaucoma; NVG= neovascular glaucoma; PEX= pseudoexfoliation; PKP= penetrating keratoplasty; POAG= primary open angle glaucoma.

* Student’s *t*-test;

** Chi-square test.

Surgery was uneventful in all cases. Scheimpflug images were obtained during follow-up examinations at POD 1 and POM 3. The perioperative data are presented in tables 2 and 3, and a comparison between the groups is shown in Table 4.

**Table 2:** Preand postoperative data of the ExPRESS group

Parameter	Preoperative	POD 1	POW 1	POM 1	POM 3	Other eye	*p-value* (Paired t-test)^*^	*p-value* (Wilcoxon)^+^
BCVA (logMAR)	0.8 ± 0.8	1.1 ± 0.7	1.1 ± 0.9	1 ± 1	0.8 ± 1	0.6 ± 0.3	0.100	0.870
1OP (mmHg)	31.9 ± 10.1	6.1 ± 5. 7	10.6 ± 4.7	14.4 ± 8.3	15.7 ± 3.6	16.7 ± 5.5	**<0.001**	**0.001**
**Pentacam** Anterior mean K	44 ± 1.7	44.1 ± 1.4	43.8 ± 1.1	43.7 ± 2	44.2 ± 1.7	44 ± 1.6	0.640	0.270
Posterior mean K	-6.3± 0.3	-6.4 ± 0.3	-6.5 ± 0.2	-6.2 ± 0.3	-6.3 ± 0.3	-6.3 ± 0.2	0.210	0.280
Anterior astigmatism	2.6 ± 3.3	4.7 ± 3.1	3.1 ± 2	3 ± 1.9	2.2 ± 1.2	2 ± 1.2	0.190	0.330
Posterior astigmatism	0.4 ± 0.2	0.9 ± 0.5	0.8 ± 0.3	0.4 ± 0.3	0.5 ± 0.4	0.3 ± 0.2	**0.008**	0.650
ACD (mm)	4.3 ± 0.7	3.5 ± 1	3.8 ± 0.9	4.2 ± 0.8	4.2 ± 0.8	4.1 ± 0.9	**0.016**	0.510
AC volume (mm^3^)	193 ± 35	160 ± 49	153.3 ± 42.8	219.5 ± 103.8	191.1 ± 33.8	189.8 ± 39.9	**0.006**	0.570
AC angle (^o^)	44.3 ± 7.3	43.5 ± 8	41.1 ± 20.8	37 ± 42.9	47.6 ± 5.3	42.8 ± 7.2	0.770	**0.025**

AC= anterior chamber; ACD= anterior chamber depth; BCVA= best-corrected visual acuity; IOP= intraocular pressure.

* Value on POD 1 vs. baseline.

+ Value at POM 3 vs. baseline. *p*<0.025 was considered statistically significant (following Bonferroni correction for two main time points, POM 1 and 3.

**Table 3:** Preand postoperative data of the trabeculectomy group

Parameter	Preoperative	POD 1	POW 1	POM 1	POM 3	Other eye	p-value (Paired t-test)^*^	p-value (Wilcoxon)^+^
BCVA (logMAR)	0.24 ± 0.29	0.64 ± 0.55	0.4 ± 0.38	0.34 ± 0.36	0.3 ± 0.44	0.15 ± 0.11	0.029	0.680
IOP (mmHg)	28.2 ± 12.4	14.1 ± 13.5	13.2 ± 5.1	13.4 ± 4.4	14.7 ± 4.7	15 ± 4.2	0.010	0.001
Pentacam								
Anterior mean K	43.7 ± 1	43.5 ± 3.1	44.5 ± 2.4	44.3 ± 0.3	43.5 ± 1.1	44 ± 1.3	0.820	0.630
Posterior mean K	-6.2 ± 0.2	-6.2 ± 0.6	-6.4 ± 0.2	-6.2 ± 0.3	-6.2 ± 0.1	-6.2 ± 0.2	1.00	1.00
Anterior astigmatism	0.9 ± 0.7	3.8 ± 3.1	3.4 ± 3.1	0.5 ± 0.3	0.9 ± 0.6	1.1 ± 0.7	0.003	1.00
Posterior stigmatism	0.3 ± 0.1	0.9 ± 0.7	0.8 ± 0.4	0.4 ± 0.3	0.3 ± 0.2	0.4 ± 0.2	0.005	1.00
ACD (mm)	3.2 ± 1.1	2.7 ± 1	3.1 ± 1.1	3.4 ± 1.1	3.3 ± 1.1	3.4 ± 0.9	0.230	0.810
AC volume (mm^3^)	140.2 ± 51.5	114.1 ± 47.3	125 ± 45.5	152.3 ± 54.8	151.7 ± 48	151.8 ± 44.2	0.190	0.560
AC angle (^o^)	36 ± 9.9	30.1 ±11.6	33.9 ± 9.3	30.3 ±11.7	33.3 ± 10	38.5 ± 10.2	0.170	0.490

AC= anterior chamber; ACD= anterior chamber depth; BCVA= best-corrected visual acuity; IOP= intraocular pressure. p<0.0125 was considered statistically significant (following Bonferroni correction for two time points).

* Value on POD 1 vs. baseline;

+ Value at POM 3 vs. baseline.

**Table 4:** Between-group comparisons of mean changes in anterior chamber parameters following surgery

Parameter	1 -day postoperative change			3-months postoperative change		
	express	Trabeculectomy *p-value* (Student’s t-test)		express	Trabeculectomy	*p-value* (Student’s t-test)
BCVA (logMAR)	**0.4 ± 1**	**0.4 ± 0.39**	**1.00**	**-0.1 ± 1.1**	**0.06 ± 0.21**	**0.54**
IOP (mmHg)	**-26.1 ± 12.3**	**-14.1 ± 17.8**	**0.05**	**-14.6 ± 12.3**	**-13.5 ± 13.4**	**0.82**
Pentacam						
Anterior mean K	**0.2 ± 1.9**	**0 ± 2.6**	**0.82**	**0.4 ± 1.2**	**-0.1 ± 0.9**	**0.19**
Posterior mean K	**-0.1 ± 0.2**	**0 ± 0.5**	**0.51**	**0.1 ± 0.3**	**0 ± 0.1**	**0.19**
Anterior astigmatism	**1.8 ± 5.1**	**3 ± 3.1**	**0.42**	**0.1 ± 1.2**	**0.1 ± 0.8**	**1.00**
Posterior astigmatism	**0.4 ± 0.5**	**0.7 ± 0.7**	**0.20**	**0 ± 0.4**	**0 ± 0.2**	**1.00**
ACD (mm)	**-0.7 ± 0.9**	**-0.4 ± 0.7**	**0.30**	**-0.1 ± 0.4**	**0.1 ± 0.6**	**0.31**
AC volume (mm^3^)	**-29.1 ± 35.2**	**-17.3 ± 37.2**	**0.38**	**-2.5 ± 16.4**	**15.9 ± 31.4**	**0.07**
AC angle (^o^)	**-0.6 ± 7.8**	**-5.1 ± 8.5**	**0.14**	**3.2 ± 5**	**-1.9 ± 8.2**	**0.06**

*p*<0.025 was considered statistically significant (following Bonferroni correction for two main time points: POM 1 and 3).

ACC anterior chamber; ACD *p*<0.025 was considered statistically significant (following Bonferroni correction for two main time points: POM 1 and 3).

AC= anterior chamber; ACD= anterior chamber depth; BCVA= best-corrected visual acuity; IOP= intraocular pressure anterior chamber depth; BCVA= best-corrected visual acuity; IOP= intraocular pressure.

In the ExPRESS group, there was no significant change in BCVA (logMAR) from baseline to either POD 1 (p=0.1) or POM 3 (p=0.87). In the trabeculectomy group, there was a transient, but non-significant, decrease in visual acuity on POD 1 (p=0.029), which resolved by POM 3 (p=0.68). No significant between-group difference in change in BCVA was found at either time point (POD 1, p=1.0; POM 3, p=0.54).

IOP decreased significantly in both groups on POD 1 (ExPRESS group, p<0.001; trabeculectomy group, p=0.01) and POM 3 (p=0.001 for both groups). Between-group comparisons showed a trend toward a greater IOP decrease on POD 1 (p=0.03), but not POM 3 (p=0.81). On POD 1, hypotony (defined as IOP <6 mmHg) was detected in 10 patients (52.6%) in the ExPRESS group and 3 (23%) in the trabeculectomy group, but none developed choroidal effusion.

Mean anterior and posterior keratometry readings of the corneas did not change significantly following surgery in either group, while there was a similar change over time between groups. An increase in anterior corneal astigmatism was noted on POD 1 in the trabeculectomy group only (p=0.003). This increase in anterior corneal astigmatism did not remain significant at POM 3 (p=1.0). When comparing the change in mean anterior corneal astigmatism between the two groups, no statistically significant difference was found at either time point (POD 1, p=0.42; POM 3, p=1.0).

Posterior corneal astigmatism increased on POD 1 in both groups (ExPRESS p=0.008; trabeculectomy p=0.005), but these changes were not evident at POM 3 (ExPRESS p=0.65; trabeculectomy p=1.0). There was a similar change in posterior corneal astigmatism at either time point (POD 1, p=0.20; POM 3, p=1.0).

The ACD decreased on POD 1, as compared to base line, in both groups, but the decrease was only statistically significant in the ExPRESS group (ExPRESS, p=0.016; trabeculectomy, p=0.23). At POM 3, there was no statistically significant change in ACD in either group (ExPRESS, p=0.51; trabeculectomy, p=0.81). No significant difference was found in the change in ACD between groups at POD 1 (p=0.30) or POM 3 (p=0.31).

The ACV decreased in the ExPRESS group on POD 1 (p=0.006), which resolved and did not remain significant at POM 3 (p=0.57). In the trabeculectomy group, ACV was decreased on POD 1 and increased at POM 3, but none of these changes were significant (p=0.19 and 0.56, respectively). There was a similar change in ACV in both groups on POD 1 (p=0.38) and POM 3 (p=0.07).

There was a small decrease in the ACA, which did not reach statistical significance, in both groups on POD 1 (ExPRESS, p=0.770; trabeculectomy, p=0.170). At POM 3, ACA remained decreased in the trabeculectomy group, though not significantly. In the ExPRESS group, there was a small, but statistically significant, increase in ACA (p=0.025). There was no significant difference in the change in ACA between the groups on POD 1 (p=0.14) or POM 3 (p=0.06).

## DISCUSSION

In this present study, the effects of trabeculectomy and ExPRESS mini shunt implantation on anterior segment (i.e., cornea and anterior chamber) parameters were prospectively evaluated with the use of the Pentacam Scheimpflug imaging system. The results revealed minor changes in anterior chamber parameters for both procedures, especially on POD 1. In our previous study^(19)^, ExPRESS shunt implantation caused a transient increase in posterior corneal astigmatism and transient decreases in ACD and ACV. These changes disappeared between postoperative week (POW) 1 and POM 3, which were attributed to transient hypotony (IOP <6 mmHg), as observed in 52.6% of eyes. We presumed that these findings could be explained by over-filtration through the ExPRESS implant. Similarly, Simsek et al., who evaluated changes in the anterior chamber following trabeculectomy based on Scheimpflug images, reported that the anterior chamber parameters were stabilized by POM 1^(21)^. They too advised that cases in which these parameters did not stabilize beyond POM 1 should be re-evaluated for over-filtration.

In the present study, there was a transient early increase in anterior and posterior corneal astigmatism in the trabeculectomy group, which disappeared between POM 1 and 3, but there was no significant change in ACD or ACV. These findings can be explained by the diffe rence in tension of the sutures placed in each group, as the sutures in the trabeculectomy group may have been tighter than in the ExPRESS group. This hypothesis can explain the greater increase in anterior astigmatism and also the lower rate of hypotony and the slightly lower decreases in ACD and ACV on POD 1 in the trabeculectomy group. However, when comparing the postoperative changes in both group on POD 1, there were no significant differences in anterior astigmatism, ACD, or ACV between the two groups (between-group analysis). ExPRESS implantation is considered by many authors to be an augmentation to conventional trabeculectomy and should not be referred to as a “true” glaucoma drainage device due to the fact that evacuation of the aqueous humor is performed at a bleb near the limbus, rather than a more posterior location. These findings indicate only minute differences between ExPRESS implantation and conventional trabeculectomy, thus supporting this notion.

In both groups, there was a decrease in IOP at both POD 1 and POM 3. The decrease in IOP trended toward being greater on POD 1 in the ExPRESS group (p=0.05). This finding may be attributed to the less tight sutures in the ExPRESS group leading to a higher rate of immediate postoperative hypotony. This difference in IOP between groups was no longer apparent at POM 3. This result is in agreement with several studies reporting no statistically significant difference between trabeculectomy and ExPRESS regarding IOP at the last follow-up examination^(2,13,22-24)^.

Interestingly, there was a trend toward a greater change in the ACA at POM 3 in the ExPRESS group. On the other hand, the postoperative ACA was consistently narrower than the preoperative ACA in the trabeculectomy group. A reduction in the ACA in the trabeculectomy group and an increase in the ExPRESS group are surprising in light of the small decrease in the ACD and ACV noted at POM 3. These findings, taking into account the high variability of the ACA parameter, warrants further investigation of this issue.

Despite the prospective design of this study, it was not absent limitations. First, the main drawback of this study is that the two study groups were not homogenous in terms of preoperative ACD and lens status (phakic vs. pseudophakic). It is routine in our department to perform ExPRESS in pseudophakic eyes due to concerns regarding potential dislocation of the device in subsequent cataract surgery based on unpublished personal correspondences. Indeed, all participants in the ExPRESS group underwent previous phacoemulsification, as compared to approximately half in the trabeculectomy group. In order to deal with this limitation, changes in the anterior chamber parameters were compared rather than absolute final outcomes. An additional limitation of this pilot study is that both groups were quite heterogeneous in terms of the type of glaucoma the subjects had, which could potentially affect the interpretation of the study results. In addition, the relatively small sample size of this pilot study may have resulted in insufficient power to detect small differences before and after surgery within and between groups. Hence, future studies should include larger sample sizes.

In summary, both ExPRESS implantation and traditional trabeculectomy significantly decreased IOP and had a transient effect on corneal astigmatism with only minor differences between the methods. Though only the ExPRESS group had a significant change in ACD and ACV on POD 1, at POM 3, this difference was no longer significant. Therefore, both procedures may be safely used with similar effects on anterior segment parameters as evaluated by Scheimpflug imaging.
